# The ZNF217 Biomarker Predicts Low- and High-Risk Oncotype DX^®^ Recurrence Score in ER-Positive Invasive Breast Cancers

**DOI:** 10.3389/fphar.2019.00524

**Published:** 2019-05-28

**Authors:** Pascale A. Cohen, Olivier Loudig, Christina Liu, Joseph Albanese, Susan Fineberg

**Affiliations:** ^1^Univ Lyon, Université Claude Bernard Lyon 1, INSERM U1052, CNRS 5286, Centre de Recherche en Cancérologie de Lyon, Lyon, France; ^2^Department of Anatomy and Structural Biology, Albert Einstein College of Medicine, Bronx, NY, United States; ^3^Center for Discovery and Innovation, Hackensack University Medical Center (HUMC), Nutley, NJ, United States; ^4^Department of Pathology, Montefiore Medical Center and the Albert Einstein College of Medicine, Bronx, NY, United States

**Keywords:** breast cancer, estrogen-receptor positive, ZNF217, expression, Oncotype DX^®^, biomarker

## Abstract

We assessed mRNA and protein expression levels of the ZN217 oncogene in 17 clinical FFPE ER-positive invasive breast cancer specimens with known (low or high) Oncotype DX^®^ Recurrence Scores. This study shows that mRNA or nuclear protein levels of the ZNF217 significantly correlate with Oncotype DX^®^ Recurrence Score.

## Report

Breast cancer (BC) is the most frequent cancer among women. Expression of Estrogen Receptor α (ERα) is found in 60–80% of BC patients, and allows an accurate prediction of response to endocrine therapy (ET). However, between 10 and 50% of ER^+^ BC treated patients will later relapse. Thus, a more precise method for stratifying patients based on their prognosis and for predicting their response to therapy remains needed.

The Oncotype DX^®^ (ODX) genomic assay tests for the expression of 21 genes and calculates a Recurrence Score (RS), which predicts the risk of distant disease recurrence in ER^+^ BC. A high RS value indicates a poor prognosis and a higher probability of distant recurrence at 10 years in patients treated with adjuvant ET (Paik et al., 2004). We have shown that high expression levels of the *ZNF217* oncogenic transcription factor are associated with poor prognosis, recurrent distant metastases and can predict response to ET in ER^+^ BC (Vendrell et al., 2012; Nguyen et al., 2014). This novel snapshot report investigates the correlation between ZNF217 expression levels (protein or mRNA) and ODX RS.

**Figure 1:**
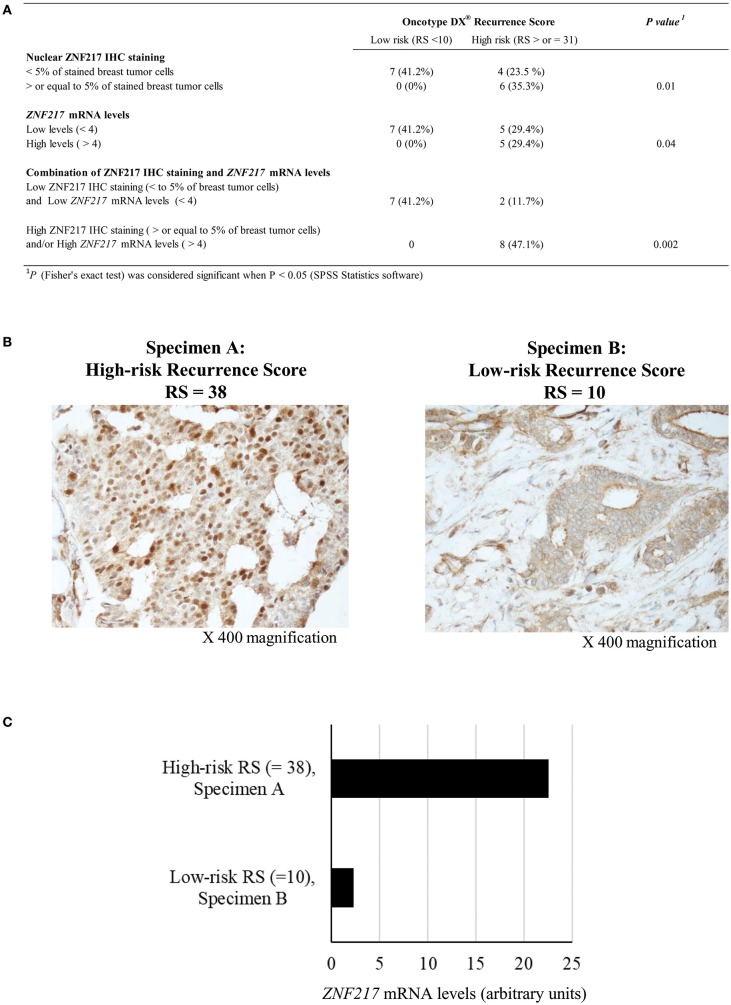
**(A)** ZNF217 predicts low- and high-risk Oncotype DX^®^ Recurrence Score. **(B)** Illustrative examples of ZNF217 IHC staining in two representative ER^+^ invasive breast carcinoma tumor samples with high-risk Oncotype DX^®^ Recurrence Score with positive nuclear staining (specimen A) or low-risk Oncotype DX^®^ Recurrence Score without nuclear staining (specimen B). **(C)** RTQ-PCR assessment of *ZNF217* mRNA levels (arbitrary units) in specimen A and specimen B.

After approval by the Institutional Review Board, the pathology database of the Montefiore Medical Center (NY, USA) was searched to identify ER^+^ BC cases with: (i) low-risk (<18) or high-risk (>31) ODX RS; (ii) sufficient tissue for both ZNF217 immunohistochemistry (IHC) (Nguyen et al., 2014) and *ZNF217* RTQ-PCR (Loudig et al., 2007; Kotorashvili et al., 2012; Vendrell et al., 2012) investigations. Seventeen FFPE clinical specimens were selected (Figure 1A). After ZNF217 IHC analysis, the percentage of positive staining of tumor nuclei was estimated (range: 0–80%). *ZNF217* mRNA levels ranged from 0.5 to 22.5 (arbitrary units) and the mean value was used as a cutoff. Figure 1 illustrates that: (i) all the clinical specimens with low-risk ODX RS displayed low *ZNF217* mRNA levels (<4) or low percentage of IHC stained nuclei (<5%); (ii) ZNF217 nuclear staining and *ZNF217* mRNA levels were significantly associated with ODX RS; (iii) combining both IHC analysis and *ZNF217* mRNA levels allowed the stratification of the samples with a better accuracy, with 100 and 80%, respectively, of low-risk ODX RS and high-risk ODX RS correctly classified and significantly association with ZNF217 expression levels (*P* = 0.002). Strikingly, two high ODX RS specimens displaying the highest *ZNF217* mRNA levels (9.2 and 22.5) also displayed the highest ZNF217 IHC staining (70–80%) and pejorative clinical record (T2 with invaded nodes and recurrent breast cancer, respectively).

Altogether, while these exploratory results were obtained in a small cohort, our preliminary data indicate a correlation between ZNF217 expression levels and ODX RS. This is in agreement with previous observations that both ZNF217 expression levels and the ODX RS are prognostic and predictive of ET response in ER^+^ BC. Supporting recent observations indicate that ZNF217 expression levels also predict neoadjuvant ET response in these patients (Vendrell et al., 2018). However, it is necessary to extend our study to a larger cohort including low-, high- but also intermediate- ODX RS specimens to investigate whether assessing ZNF217 levels (alone or in combination with ODX) could provide additional information to the current well-established ODX genomic assay.

## Ethics Statement

The protocol was approved by the Institutional Review Board, Montefiore Medical Center and Albert Einstein College of Medicine (NY, USA).

## Author Contributions

PC and SF conceived the study. SF supervised and analyzed the IHC experiments performed by JA. OL supervised and analyzed the RTQ-PCR experiments performed by CL. PC and SF co-analyzed the data. PC wrote the manuscript.

### Conflict of Interest Statement

SF served in an expert advisory panel for Genomic Health. The remaining authors declare that the research was conducted in the absence of any commercial or financial relationships that could be construed as a potential conflict of interest.

## Abbreviations

BC: Breast Cancer
ERα: Estrogen receptor α
ER+: Estrogen receptor α-positive
ET: endocrine therapy
ODX: Oncotype DX^®^
RS: Recurrence Score
FFPE, Formalin-Fixed: Paraffin-Embedded
IHC: immunohistochemistry
RTQ-PCR: real-time quantitative polymerase chain reaction.
